# Monitoring the impact of decentralised chronic care services on patient travel time in rural Africa - methods and results in Northern Malawi

**DOI:** 10.1186/1476-072X-11-49

**Published:** 2012-11-15

**Authors:** Rein MGJ Houben, Thomas P Van Boeckel, Venance Mwinuka, Peter Mzumara, Keith Branson, Catherine Linard, Frank Chimbwandira, Neil French, Judith R Glynn, Amelia C Crampin

**Affiliations:** 1 Karonga Prevention Study, Chilumba, Malawi; 2 Department of Infectious Disease Epidemiology, London School of Hygiene and Tropical Medicine, London, United Kingdom; 3 Biological Control and Spatial Ecology, Université Libre de Bruxelles, CP 160/12, Avenue FD Roosevelt 50, Brussels, B, 1050, Belgium; 4 Fonds National de la Recherche Scientifique (F.R.S.-FNRS), Rue d’Egmont 5, Brussels, B, 1000, Belgium; 5 Karonga District Hospital, Karonga, Malawi; 6 Ministry of Health – HIV/AIDS unit, Lilongwe, Malawi; 7 Institute of Infection & Global Health, University of Liverpool, Liverpool, United Kingdom

## Abstract

**Background:**

Decentralised health services form a key part of chronic care strategies in resource-limited settings by reducing the distance between patient and clinic and thereby the time and costs involved in travelling. However, few tools exist to evaluate the impact of decentralisation on patient travel time or what proportion of patients attend their nearest clinic. Here we develop methods to monitor changes in travel time, using data from the antiretroviral therapy (ART) roll-out in a rural district in North Malawi.

**Methods:**

Clinic position was combined with GPS information on the home village of patients accessing ART services in Karonga District (North Malawi) between July 2005 and July 2009. Potential travel time was estimated as the travel time for an individual attending their nearest clinic, and estimated actual travel time as the time to the clinic attended. This allowed us to calculate changes in potential and actual travel time as new clinics opened and track the proportion and origin of patients not accessing their nearest clinic.

**Results:**

The model showed how the opening of further ART clinics in Karonga District reduced median potential travel time from 83 to 43 minutes, and median actual travel time fell from 83 to 47 minutes. The proportion of patients not attending their nearest clinic increased from 6% when two clinics were open, to 12% with four open.

**Discussion:**

Integrating GPS information with patient data shows the impact of decentralisation on travel time and clinic choice to inform policy and research questions. In our case study, travel time decreased, accompanied by an increased uptake of services. However, the model also identified an increasing proportion of ART patients did not attend their nearest clinic.

## Introduction

Poor transport infrastructure and scattered populations in low income countries force people from rural communities to spend a significant amount of their time and scarce income in travelling to meet basic needs such as healthcare [1,2], which has been shown to be a barrier to initiating care [3]. A key component in providing essential healthcare in these settings is decentralised services, which reduces the distance a patient has to travel to a clinic, and thereby travel time and costs. The central tenet of decentralisation is that proximity of health services will remove a key barrier to care, thus increasing the numbers accessing and initiating care, as well as improve retention in case of long-term or life-long treatment, for example antiretroviral treatment (ART) or treatment for Non-Communicable Diseases (NCDs) like diabetes and cardiovascular diseases [4,5].

However, few tools exist to examine the impact of decentralisation on patient travel time. Using Geographical Information Systems one can link the area of residence with the clinic accessed for their care [6,7], and assess how health service utilisation changes as clinics closer to home start providing the required care. This will help evaluate the impact of decentralised services based on one of its key metrics, the travel time.

In Malawi, one of the poorest countries in the world, the number of people receiving ART expanded from 10,000 in 2004 to 200,000 in 2009 [8], with immediate impact on mortality [9,10]. As laboratory facilities are not a prerequisite, the Malawi programme was able to rapidly decentralise its ART services [11]. ART was first established at the main central hospitals, and then expanded to the regional District hospitals, followed by further decentralisation in strategically chosen clinics within districts to improve patient accessibility.

In this paper we present methods that can monitor and visualise changes in travel time as services decentralize, using data from the roll-out of ART services in a rural district in North Malawi for illustration.

## Data and methods

### Setting - Karonga District

**Figure 1:**
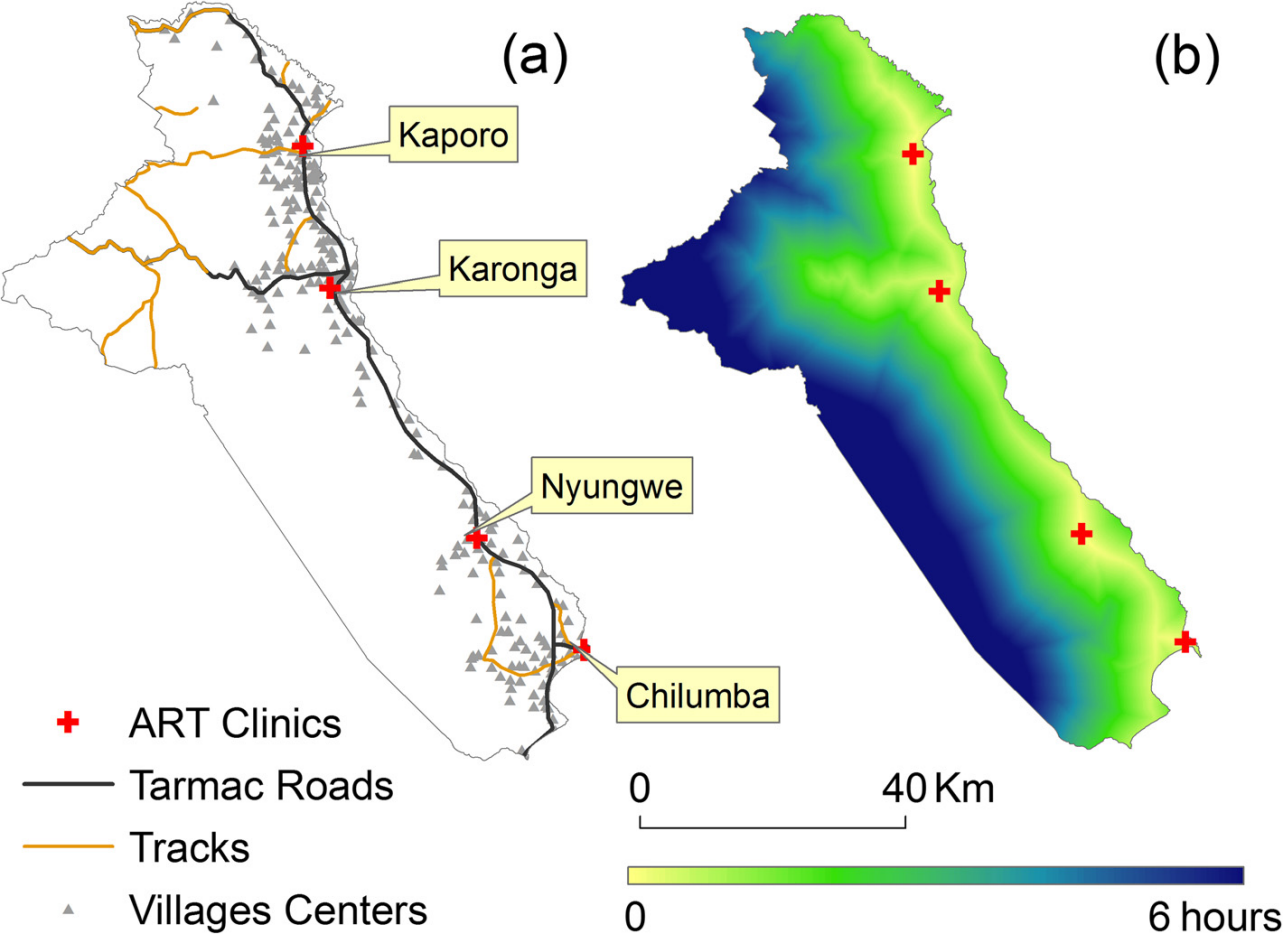
**a-b: Karonga District with main roads, villages and ART clinics (a), and travel time surface to the nearest ART facility in January 2008 (b) KRH = Kaporo Rural Hospital; KDH = Karonga District Hospital; NHC = Nyungwe Health Center; CRH = Chilumba Rural Hospital.** Population density derived from 1998 population census of Malawi [13]. The West of the district is sparsely populated areas and village boundaries become very extended in this area, which is the reason no village centre points are located there.

Karonga District covers an area of 3355 km^2^ and is bordered by Tanzania to the north, a mountain range to the west and south and Lake Malawi to the east. The population is ~270,000, with the majority of people living close to the lake (see Figure 1a - villages are shown in grey triangles). The majority of the population are subsistence farmers, fishermen or small traders. Since 1979 the Karonga Prevention Study has been conducting health research in the district, and has developed a system of village documentation that is in line with the traditional authority structure of village headmen [12]. The system is updated continuously as village boundaries are not static. For the majority of these villages the GPS coordinates of a central point is recorded in the project database. Relative population size estimates for each village were derived from the 1998 Malawi census [13] as data from the more recent 2008 census was not available in sub village level to allow recoding into KPS village codes. The database also holds data on the road network (held as vector data), which includes all tarmac roads and main tracks that could be traversed in a saloon car.

### ART roll-out in Karonga District

The first ART clinic in Karonga District opened in July 2005 at Karonga District Hospital, followed by the clinic at Chilumba Rural Hospital in the south of the District in 2006, and 2 further clinics in the central (Nyungwe Health Centre) and northern area (Kaporo Rural Hospital) in 2008 (Figure 1a). As Figure 1a illustrates, the opening of each new clinic was chosen strategically to maximize the reduction of potential travel time, each servicing a different cluster of villages.

### Data collection - ART patients

We collected demographic, ART history and outcome information for all adults (15 years or older) who had received ART from the clinics in Karonga District up to August 2009, through anonymised digital photographs of patient registers from each clinic. Village of residence was recorded as free text from the ART register and linked to the nearest village in the project database that had GPS coordinates available. All data were double entered and checked.

We calculated the number of patients receiving ART in Karonga District at the end of each calendar year and the end of follow up (July 2009), as well as the proportion who had started ART at their current clinic.

### Travel time surface

The GPS information was processed in a cost surface approach to derive the travel time between any village in Karonga District to ART facilities [14]. The spatial resolution of the analysis was set to 100mx100m. Three classes were defined on the cost surface: i) tarmac roads (speed = 35 Km/h), ii) tracks (speed = 5 Km/h), iii) any other land types: settlements, crops, ways, bare areas (speed = 3.5 Km/h). The location of the main tracks and tarmac roads was integrated in the cost surface after conversion to 100 m by 100 m pixels by applying a buffer zone of 75 meters on both sides of the roads.

The speed on a tarmac road was estimated from reports from experienced project staff as the average speed of a minibus operating along the main road (accounting for bus stops and police and customs check points). Travel speed on tracks reflects the preference for travelling along major tracks rather than small paths, accounting for the possibility of paying for a ride on a motorized vehicle or spending a smaller amount of money on a bicycle taxi. The value of 3.5 Km/h chosen for all remaining routes reflects the average walking speed of a patient travelling in a straight line to the hospital. [14] Absolute walking speed may be higher than these values, but walking speeds are expressed as straight lines towards the destination (road or clinic). In reality, although small paths and tracks are ubiquitous across all surfaces they do not necessarily run in straight lines and derived walking speeds take this effect into account. In sensitivity analyses we explored the impact of a range in travel speeds for each surface.

Average travel time to an ART clinic for the general population was calculated using each village, weighted by village size in the census. This was recalculated following the opening of each new ART clinic.

Using the travel time surface, we estimated the actual travel time for each ART patient from their village to their current ART clinic in each quarter. The route was chosen according to the least cost path (smallest cumulative time from clinic to village) this route would in most case lead to the use of tarmac preceded by either track or path according to which one led to the trajectory minimizing to total travel time. We also estimated the potential travel time, if they had travelled to the clinic nearest to their village. We mapped the median actual and potential travel times for the population to visualise the difference.

Comparing potential and actual travel time, we identified patients not attending their nearest clinic. We explored whether these patients were more likely to transfer to another (probably closer) clinic. Two time periods were compared, September 2006 to January 2008 (when a patient could either attend Chilumba or Karonga clinic), or February 2008 to end of study. Patients were categorised attending as attending their nearest clinic or not based on clinic attended at the start of the study period or start of ART. Whether they then later transferred was extracted from the outcome status.

Cost surfaces used to derive average travel times to ART facilities were generated with ArcGIS 9.3 *Spatial Analyst* ( http://www.esri.com). The time series analyses were performed with the open access statistical computing software R (cran.r-project.org/) using the package *maptools*. Risk ratios were calculated using log binomial regression, differences between the two time periods were explored using the likelihood ratio test. Statistical analyses were done using Intercooled Stata version 11 ( http://www.stata.com).

### Ethics statement

The research was approved by the National Health Sciences Research Committee in Malawi (NHSRC#424) and the Ethics Committee of the London School of Hygiene and Tropical Medicine (LSHTM#5067). As all data was recorded from anonymised clinic records, and is presented in an aggregated format, no individual patient consent was needed. Permission to use the anonimised patient registers was given by national, regional and clinic ART coordinators.

## Results

### General ART accessibility Karonga (whole population)

A total of 5575 ART client records were recorded in the database. Based on the free text village description we were able to assign a KPS village code for 97% of these (5411/5575). Out of the 353 village codes used, less than 9% (29/353) had GPS coordinates missing and were merged with the nearest village with GPS coordinates.

**Figure 2:**
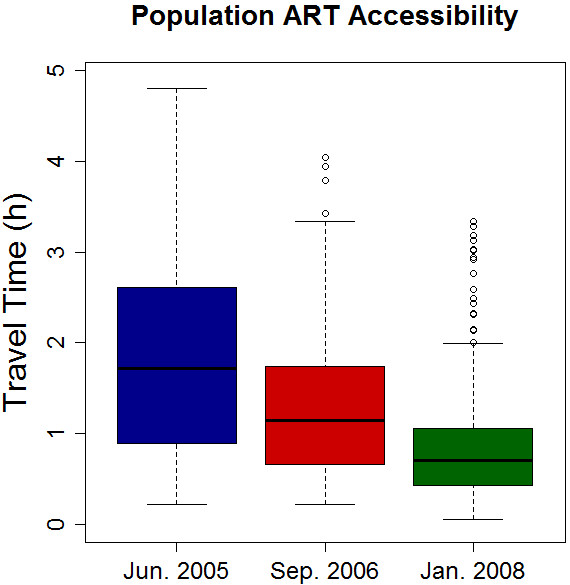
**Potential travel time over time.** Box plots show distribution of average travel time for the general population to the nearest ART clinic. Travel times estimated using travel time surface in Figure 1.

Figure 1b illustrates the how the model estimated the duration of travel for the general population from any point in Karonga District to their nearest ART facility after the third and fourth clinics started delivering ART in January 2008. The impact of decentralisation of services on this travel time is illustrated in Figure 2. Under current model assumptions, the median potential travel time to the nearest ART clinic for any person living in Karonga District was almost halved (from 1 h 23 min to 0 hr 43 min). For the 50% of the population living furthest away from a clinic (the top half of the box plots) the model estimated it was reduced by around two thirds (from 2 hr 36 min to 1 hr 03 min). Similarly, distance to clinics was reduced from 25.7 Km (median) in July 2005 to 6 Km after the opening of further clinics.

### Patient response to improved accessibility

**Figure 3:**
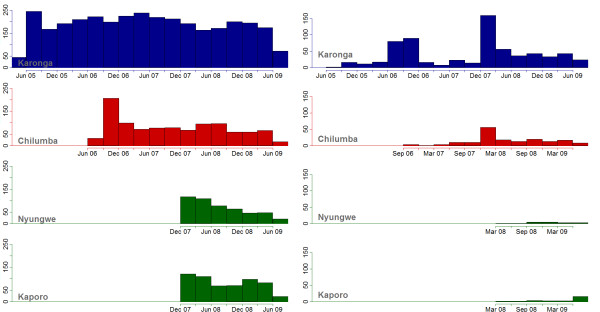
a-b: Total number of people starting at each clinic each quarter (a - left) and total number of people transferring out each quarter (b - right).

Figure 3a shows the number of people starting at each clinic throughout the study period. The spikes of transfers out from existing clinics (Figure 3b) correspond to the opening of a new clinic, and illustrate patient mobility, i.e. that many may transfer to another (probably) nearer clinic when it becomes available. Excluding people who transferred in, the average number of people starting ART each month in the district increased from 56 (standard deviation (SD) =10) when only the Karonga clinic had opened, to 82 (SD = 16) after the opening of Chilumba clinic and 104 (SD = 15) when Nyungwe and Kaporo Clinic opened.

### Potential versus actual travel time (ART patients)

**Figure 4:**
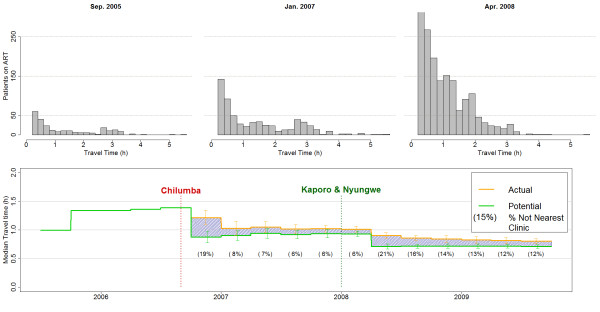
**a-b: Distribution of actual travel time for ART receiving population (a - top), Median actual (orange line) and potential (green line) travel time (b - bottom).** Travel times estimated using travel time surface in Figure 1. Note Figure 4b: The green line shows Potential Travel Time (PTT) if all people receiving ART went to their nearest clinic. The orange line shows the estimated Actual travel Time (ATT). Error bars show 95% confidence intervals for the median (using bootstrapping). The proportions below the green line indicate the proportion of patients not attending their nearest clinic.

Figure 4a shows the distribution of actual travel times for patients attending an ART clinic. Each was calculated 3 months after new clinics opened, to incorporate the high numbers transferring to their nearer clinic. It illustrates the continuous increase uptake of ART services over time. After the clinics at Kaporo and Nyungwe started delivering ART services, the proportion of ART patients whose actual travel time was less than 1.5 hours increased strongly.

For patients receiving ART the median potential minimum travel time to the nearest ART clinic decreased sharply with the opening of the Chilumba clinic (Figure 4b - green line), and decreased again when Karonga and Nyungwe clinics were opened. The slight increases in the potential travel time before each new clinic opened are due to patients from increasingly remote villages accessing ART. The persistent difference between actual and potential travel time illustrate that a proportion of the ART population does not attend their nearest clinic which is shown in Figure 4b (below green line). These indicate that after the 2 last clinics opened in 2008, a higher proportion of patients persisted with attending a not the nearest clinic, although ‘extra’ travel time per patient was smaller.

The analysis showed that patients not attending their nearest clinic were more likely to transfer out in both time periods (see Table 1). However, the association became less pronounced during the second period (Risk Ratio (95% CI) = 3.11 (2.72-3.56) vs. 2.30 (1.94-2.73), p-value Likelihood Ratio test for interaction = 0.007). Also, the proportion of patients who transferred dropped strongly.

**Table 1:** Association between attending nearest clinic and transferring out

**Time period**		**Transferred % (n/N)**	**Risk Ratio (95% CI)**
Sep 06- Jan 08	Not in nearest clinic	65.1 (121/186)	3.11 (2.72-3.56)
	In nearest clinic	20.9 (418/1998)	
Feb 08 – Aug 09	Not in nearest clinic	23.8 (145/610)	2.30 (1.94-2.72)
	In nearest clinic	10.3 (370/3577)	

P-value likelihood ratio test for difference between time periods = 0.007.

### Sensitivity analyses

Similar trends were seen when we explored the impact of different travel speeds; (walking speed between 3 and 6 km, track road speed between 5 and 8 km/h and tarmac road speed between 30 and 45 km), but although the absolute values for potential and actual travel time were affected, the observed trends were similar.

## Discussion

We combined geographical information on area of residence and clinic use to monitor the impact of decentralised chronic care services on estimated patient travel time, illustrated by a case study of ART roll-out in Malawi. A marked reduction in both potential and actual travel time was observed. However, the methods also highlighted that a growing proportion of patients does not attend their nearest clinic, and patients are actually less likely to transfer to another clinic as more available.

This work had several limitations. We did not have direct information on transport type which prevented us from using a hybrid walking-public transport model as presented in Tanser et al. [14]. The travel speeds across surfaces were estimated from literature and local expert knowledge. As is likely to be the case other settings, budgetary and logistical restrictions stopped us from conducting the necessary field experiments. Fortunately, the sensitivity analyses used in these methods showed that our results were robust to variations in assumed travel speeds. The travel time surface was made using a number of simplifications. Firstly, we did not include seasonality and the impact of rivers being easy to cross during the dry season, and often impossible to cross during the rains. However most “track” journeys are parallel to the rivers, which run perpendicular to the tarmac road, and then along the road, so the effect of rivers would be limited. Altitude was left out as well, but as >98% of the population lives on the lakeshore or the plains, we feel this is a reasonable step to take. Using centre point of villages rather than the exact location of households reduces the precision of the estimates. Some villages can be quite spread out, especially further away from the main roads. However, village-level estimates are reasonable for the purposes of the analyses presented here. It is possible that ART patients systematically misreport where they live (to claim closer residence to a preferred clinic), but we have no evidence of this. We assumed an average travel time for each surface, although it is possible that some people choose to walk alongside the tarmac rather than travel with a minibus, particularly for shorter distances. This means that our estimated travel times could be slightly underestimated for those individuals.

These data included 4 clinics in 1 rural district, but these methods can easily be expanded to include a larger area and time span. The methodological framework can be generalised to other settings, where patient data on area of residence can be collected during clinical visits [6] to fit with public resources that have GPS-coordinates available, e.g. through census enumeration areas, as is the case in Malawi. Clinic locations are available at central registries.

These methods allowed us to study the process of decentralisation of health services in a rural district in sub Saharan Africa, and visualise how it affected patient travel time. The strong reductions in potential and actual travel time, accompanied by increasing numbers of new patients starting ART, illustrate the value of decentralisation for getting patients started on therapy. Our approach also identified patients who are not attending their nearest clinic and how their behavior changed as services continued to become decentralised.

Results from KwaZulu Natal showed how distance to nearest clinic was strongly correlated with uptake of ART [15], which seems to be confirmed by the increase in patients starting ART as more (decentralised) clinics opened. Some of the patients who do not attend their nearest clinic may wish to remain at the clinic where they initiated ART. As the initial clinics were located at the larger hospitals this preference may be related to the perceived level of available care (availability of CD4 counts or drugs for opportunistic infection) or the relative anonymity of a larger clinic. Qualitative research of ART uptake at Chilumba Rural Hospital suggests patients may wish to hide their status in their home village or seek care from a preferred clinician [16]. A study in a Southern District found that barriers other than distance to clinic (e.g. fear of drug supply problems), were important reasons for TB patients not to start ART [17].

In Sub Saharan countries the question of how to deliver care that is widely needed in challenging settings is important, [18] as ART services expand but resources reduce and decentralised care for other chronic conditions will also become more prevalent [4,5]. Researchers and policy makers need tools to better understand how decentralised services impacts on patient care seeking behavior, including uptake, revision. The methods presented here provide useful tools to better understand the impact of decentralisation on patient’s travel time to clinic and should be used as a platform to address further questions.

## Competing interests

All authors declare that they have no conflicts of interest.

## Authors’ contribution

RH and TvB had the idea for the study, RH, KB, VM and AC collected the data, TvB developed the model with contributions from CL, PM and FC provided materials, RH and TvB wrote first draft of manuscript, to which NF, JG and AC contributed. All authors read and approved the final manuscript.
